# Effect of Different Shear Rates on Particle Microstructure of Cementitious Materials in a Wide Gap Vane-in-cup Rheometer

**DOI:** 10.3390/ma13092035

**Published:** 2020-04-27

**Authors:** Mahmoud Eslami Pirharati, Hans-W. Krauss, Carsten Schilde, Dirk Lowke

**Affiliations:** 1 Institute of Building Materials, Concrete Construction and Fire Safety, Technische Universität Braunschweig, 38106 Braunschweig, Germany; h.krauss@ibmb.tu-bs.de (H.-W.K.); d.lowke@ibmb.tu-bs.de (D.L.); 2 Institute of Particle Technology, Technische Universität Braunschweig, 38106 Braunschweig, Germany; c.schilde@tu-bs.de

**Keywords:** rheology, cementitious suspension, agglomeration, microstructure, shear-rate, FBRM

## Abstract

Rheological properties of cementitious suspensions are affected not only by their mixture composition but also by process-related factors such as shear history. To enable a model-based description, investigations were carried out on the effect of shear history (shear rate variation over time) on the cement paste agglomeration state. Therefore, a Focused Beam Reflectance Measurement (FBRM) system and a wide gap rheometer were coupled to study the relation between shear history and in-situ chord length distribution simultaneously, indicating particle agglomeration. Hence, the effect of average shear rates (resulting from the applied shear profile), as well as shear rate distribution within the gap (local shear rates) on the particle agglomeration state have been investigated. The rheological properties of cement paste were evaluated with the Reiner–Riwlin approach. Furthermore, the agglomeration state of the particles was compared for different average shear rates and local shear rates at various positions of the FBRM probe. The results show that the median chord length increases in all positions when the average shear rate is decreased, indicating increasing particle agglomeration. Moreover, due to variable local shear rates at different FBRM probe positions, different agglomeration states are observed, resulting from two factors, shear rate dependent particle agglomeration and shear-induced particle migration.

## 1. Introduction

The characterization of the rheological properties of cement pastes as a function of different influencing factors, such as mixing intensity, shear history, chemical and physical properties, and microstructure has been in the focus of research within the last decades [1,2,3]. Due to the alterations of the particle microstructure (e.g., particle agglomeration) under different shear rates, resp. shear histories (shear rate variations over time) the rheological properties of cement paste can change significantly [4,5,6,7,8]. Depending on the actual microstructural properties, particle characteristics and mix composition, different types of macro flow behavior of cement paste can be identified, such as shear thinning, linear or shear thickening behavior [1,5]. To enable proper modeling of rheological properties as a function of shear history, in-depth investigations are required to link the shear rate, microstructure, and rheology of cement paste. In recent studies, an increasing focus was set on the effect of the aforementioned influencing factors on rheological properties [1,2,3,6,9]. In particular, first results on the relation between the in-situ particle size distribution and the rheological properties of cement paste have been published [1,2,10,11,12,13,14,15].

A variety of techniques are capable of determining the microstructure of cementitious materials in-situ, i.e., under shear conditions. As an example, the Focused Beam Reflectance Measurement (FBRM) system is widely used for measuring the in-situ chord length distribution. Yang et al. [15] integrated a FBRM system into a wide gap rheometer to investigate the complex rheological behavior of cement paste backfill (CPB) at different shear rates. It was figured out that the rheological behavior of CPB is not always constant with increasing shear rate (changing from shear thinning to shear thickening behavior), accompanied by changes in chord length distributions and hence, different agglomeration states. Based on the results, an increase of shear rate from 0 to 150 s^−1^ effects a decrease of the mean chord length. Qian et al. [13] investigated the effect of polycarboxylate ether superplasticizer (PCE) on flocculation state, dynamic yield stress, and thixotropy of cement paste in terms of chord length distribution of the cement particles using a coupled FBRM-rheometer. In this investigation, the time-dependent chord length distribution of cement paste was determined under a high constant rotational velocity (600 rpm). Over time, the counts of agglomerates with chord lengths smaller than 40 μm increased, while those larger than 40 μm decreased, representing a breakdown of larger agglomerates into smaller agglomerates over constant high shearing. Ferron et al. [2] investigated the effect of shear history on agglomeration and structural breakdown kinetics of the paste matrix. The results revealed a decrease of mean chord length of about 11% at a constant rotational velocity (ω = 400 rpm), indicating a disagglomeration of particles. Furthermore, the effect of different shearing regimes on the breakdown and rebuilding states of flocculation was discussed. Based on the results, the time scale to recover the structure was longer compared to structural breakdown (the kinetics of re-agglomeration are relatively slow) under the same shear regime. Haist [9] developed a material law to describe the deformation behavior of cement suspensions under consideration of the physical interactions between individual cement particles. According to his work, agglomerate structures are constantly being formed and immediately destroyed when shearing initiates. Moreover, if the shear stress is further increased, the destruction of agglomerate structures will increasingly occur in addition to new formations. Han et al. [1,6] studied the influence of high mixing intensity on microstructure, rheology, and hydration of cement paste. It was figured out that the apparent viscosity of the cement paste was increased by increasing solid volume fraction. Furthermore, a larger mean chord length (indicating agglomeration) and a higher apparent viscosity of paste were observed in samples prepared with very high mixing intensity (4000–12000 rpm). Krauss et al. [12] determined the relationship between rheological properties of suspensions and their microstructure using a 3D Optical Reflectance Measurement (3D ORM) probe in a wide gap rheometer. The effect of different mixing regimes on microstructure and the apparent viscosity of suspension were investigated. It was shown that the median particle (agglomerate) size at lower mixing intensity is clearly larger than at higher mixing intensity, hence, the strong effect of different agglomeration states on apparent viscosity of the suspension could be proven. Kim et al. [10] investigated the influence of ground-granulated blast-furnace slag on the rheological properties of cement paste in a plate–plate rheometer. Beside the rheological measurements, cement powder particle size distribution and fresh state microstructural measurements were conducted using three different techniques; a coupled stroboscope-rheometer, a conventional laser diffraction technique and a coupled laser backscattering-rheometer (in-situ). Based on these results, the in-situ measurement via a laser backscattering system revealed a higher median chord length of the cement paste compared to the median particle size determined by laser diffraction for a cement powder. The main reason is that the laser backscattering system is measuring and reporting the agglomeration state (in dense suspensions); while the laser diffraction technique can only detect the particle size distribution of the cement powder diluted in alcohol (the sample is deflocculated and the reported size corresponds to a single particle). Furthermore, the laser backscattering results were compared at low (1 s^−1^) and high (150 s^−1^) shear rates, and it was concluded that with increasing mixing intensity the size of agglomerates within the paste systems decreased.

## 2. Materials and Methods

### 2.1. Aim and Concept of Investigations

The main objective of the present contribution is the investigation of the in-situ agglomeration state under different average shear rates (resulting from the applied shear profile) and local shear rates (at different radial positions within the gap). In order to accomplish this goal, a FBRM system was integrated into a wide gap vane-in-cup rheometer to enable simultaneously the investigation of the relationship between shear rate, rheological properties and particle microstructure (agglomeration state) of cementitious materials. From the results, the rheological properties of cement pastes were determined with the Reiner–Riwlin approach. Additionally, the plug flow radii at different average shear rates were calculated with an analytical approach and numerically by Computational Fluid Dynamics (CFD) simulations. Simultaneously, the chord length distributions measured by FBRM were evaluated for different average shear rates at three different radial positions over gap. Finally, the chord length distribution affected by different local shear rates in the individual radial positions of the FBRM probe are compared and discussed.

It is important to add that a reliable shear rate distribution over gap during rheometer testing is required to better understand and compare chord length distributions detected by the FBRM probe at different radial positions. Therefore, CFD simulations were carried out as preliminary work to determine the exact nonlinear shear rate distributions over radial direction as well as over height in a wide gap rheometer for two different Newtonian fluids [16]. Based on these findings, CFD simulations were extended to determine the shear rate distributions within the gap for the cement paste investigated in order to take into account factors (e.g., stress peaks) that are not considered in the analytical calculations. As a result, the impact of local shear rates (at different radial positions) on the agglomeration state can be analyzed and compared more precisely.

The results in this paper are important for a deeper understanding of the influence of shear rate variation on the microstructure and provide the basis for the development of a model linking shear rate, agglomeration state, and rheological behavior of cement paste.

### 2.2. Materials and Sample Preparation

Ordinary Portland Cement CEM I 42.5 R (OPC; HeidelbergCement) was used in this work with a density of 3.12 g/cm^3^, and a specific surface area of 3615 cm^2^/g measured by the Blaine method. The mean particle size d_50_ of the cement was 14.8 μm. The clinker phase composition was 55.8% C_3_S, 14.6% C_2_S, 10.9% C_3_A, and 7.4% C_4_AF. More data on the chemical and physical characteristics of the cement can be found in [17]. The cement paste consisted of cement and demineralized water, only (no superplasticizer), with a water-to-cement ratio of 0.44 (volumetric solid fraction of 0.42).

Quantities of 1698 g cement (stored at 20 °C) and 754 g demineralized water (cooled at 10 °C) were mixed in a mortar mixer, according to DIN EN 196-1 [18], resulting in 1.3 dm^3^ of cement suspension. The mixing procedure with individual steps and duration is given in Table 1. The temperature of the raw materials was controlled to achieve a temperature of the fresh cement suspensions measured immediately after mixing in the range of 20 ± 0.5 for all samples.

### 2.3. Experimental Setup and Program

Three different standard rheological tests were carried out to characterize cement paste before starting the rheometer tests coupled with the FBRM system (Particle Track^TM^ G400, Mettler Toledo [19]). Firstly, the bulk density of the cement paste was determined according to DIN EN 1015-6 [20] for each sample. Secondly, a sedimentary stability test according to DIN EN ISO 10426-2 [21] was performed to verify the sedimentation stability of the sample. Then, the sample was remixed at speed level 2 for 60 s. Thirdly, the spread flow of the cement paste was determined using a Haegermann cone (d_1_ = 70 mm, d_2_ = 100 mm, h = 60 mm, according to DIN EN 1015-3 [22]) on a dry glass flow table. Simultaneously, the cup of the rheometer was filled with cement paste. The Haegermann cone was lifted at the same time as the rheometer experiment was started. It should be noticed that the time between the end of remixing (at speed level 2 for 60 s) and the start of the rheometer experiment was exactly 60 s.

**Figure 1:**
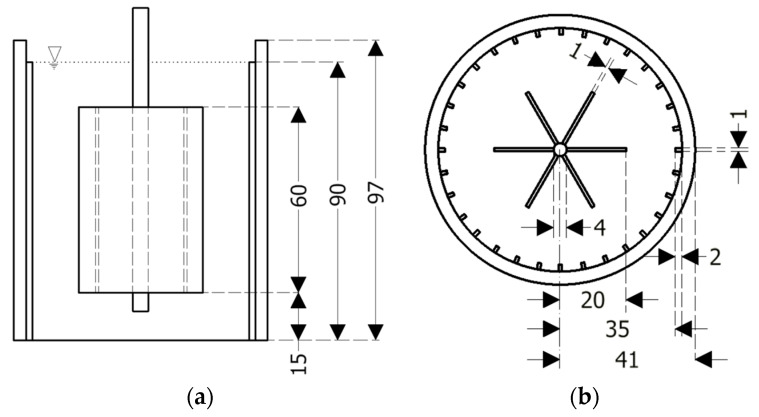
Scheme of the vane-in-cup rheometer setup with 24 vertical lamellae fixed at the outer stationary cylinder and a six-blade vane rotor; (**a**) vertical cut, (**b**) top view; all dimensions in mm.

The rheological experiments were conducted in a wide gap vane-in-cup rheometer (Anton Paar MCR 502), coupled with the FBRM system. The rheometer has a stationary outer cylinder and a rotating vane probe, where torque is recorded (Searle type rheometer). To eliminate wall slip, 24 vertical lamellae were fixed at the outer wall. The details and dimensions of the rheometer setup are displayed in Figure 1.

**Figure 2:**
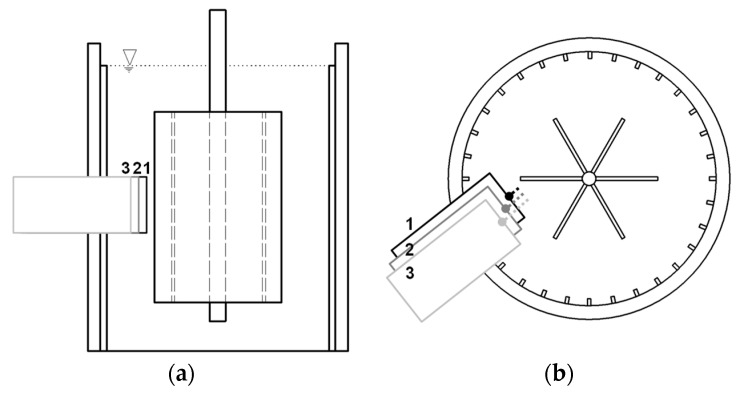
Scheme of the FBRM probe integrated into the rheometer at three different radial positions (the distance of each position from the middle point until the tips of the vane probe is shown as dash lines), each with an angle of 45° between the probe window and the flow direction; (**a**) vertical cut, (**b**) top view.

The FBRM system was integrated into the rheometer as shown in Figure 2 to characterize the chord length distributions (CLD) [1,2,15,19]. The chord length is the distance from one side to the opposite side of a particle or agglomerate detected by a highly focused laser beam rotating at a constant speed and recorded as a signal by FBRM. The time interval of the laser scanning one particle is evaluated, and the chord lengths are calculated by multiplying the scan speed by the time interval (chord length = v_laser_ ∆t) [2,11,15,19,23]. The FBRM probe diameter was 19 mm, the laser rotation diameter was 4.83 mm, the scan speed was 2 m/s, and the signal of the FBRM probe for a complete CLD was recorded every 2 s.

The experiments were performed with the FBRM probe located at three different radial positions (distance of 3.5, 5.5, and 7.5 mm from the tips of the inner vane probe, as shown in Figure 2) to determine the chord length distribution of cement paste as a function of radial position. In each position, the probe window had an angle of 45° to the flow direction to ensure proper results. Each experiment was repeated three times, yielding proper repeatability.

**Figure 3:**
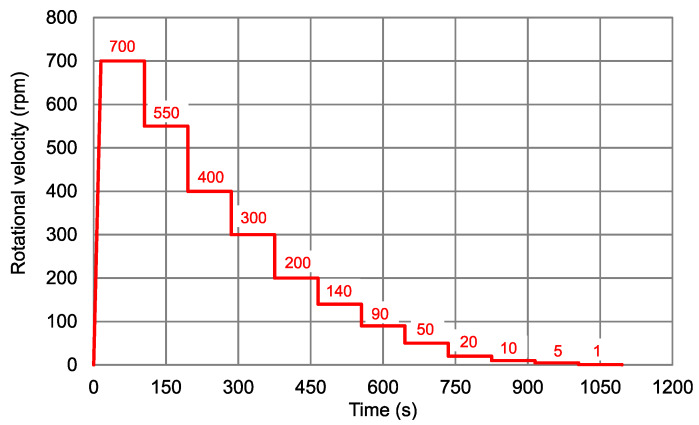
Rotational rheometer profile consisting of different steps of rotational velocity.

For the tests, the rotational velocity profile given in Figure 3 was used. Firstly, the rotational speed was increased from 0 rpm to 700 rpm within 15 s (ramp). Then, the rotational speed was decreased stepwise, each step lasting for 90 s, see Figure 3.

### 2.4. CFD Simulations

3D numerical simulations of the flow behavior of the cement paste investigated in a wide gap vane-in-cup rheometer (Searle type) have been carried out to determine the actual shear rate distribution over the gap using the commercial software ANSYS Fluent Academic Research 18.0. Based on the results discussed in [16], it was concluded that the reliable shear rate distributions within the shear gap in a wide gap rheometer can be accurately determined by numerical simulations considering specific effects, such as stress peaks on vane tips, whereas such effects are not taken into account by analytical models.

The Multiple Reference Frame (MRF) Model was used to enable rotating the vane probe at different rotational velocities, while the outer cylinder was stationary. No-slip boundary conditions were defined at all walls. Tetrahedral elements were used to discretize the geometry 3D domain. Furthermore, the non-Newtonian Herschel–Bulkley model was chosen for the simulations with flow index n=1 and the consistency index $K=\mu_{p}$, representing the Bingham model. The CFD simulations were performed for different rotational velocities (10–300 rpm) to determine i) the shear rate distribution over the gap, and ii) the plug flow radius. The results allow for comparing and discussing the variations in median chord length due to the different local shear rates at different radial positions of the FBRM probe within the shear gap.

### 2.5. Data Evaluation

#### 2.5.1. Bingham Model

To determine the rheological properties from experimental data (torque vs. rotational velocity), the Bingham model was used. The Bingham model has been frequently used to approximate the flow curves of cement paste due to the fact that most cement pastes exhibit clear yield stress and a partially linear visco-plastic flow regime. It should be noted that the linear Bingham model is an acceptable approximation over a range of low shear rates for most concrete mixtures [24,25]. The rheological parameters, yield stress, and plastic viscosity, are determined as follows by the Bingham model [24]:

(1) $$\{\begin{array}{cc} \tau =\tau_{0}+\mu_{p}\dot{\gamma } & \left|\tau \right|>\tau_{0}\\ \dot{\gamma }=0 & \left|\tau \right|\leq \tau_{0} \end{array}$$

where $\mu_{p}$ is the plastic viscosity, $\tau_{0}$ is the yield stress, τ is the shear stress, and $\dot{\gamma }$ is the shear rate.

One of the main challenges in evaluating wide gap rheometer data is the conversion of experimental data (torque, rotational velocity) to rheological parameters (shear stress, shear rate), which is due to the nonlinear shear rate distribution over the gap. Therefore, the Reiner–Riwlin approach was used to calculate the Bingham parameters in the wide gap rheometer, taking account of plug flow. The plug flow radius can be calculated as follows [24,26,27,28]:

(2) $$r_{\mathrm{plug}}=\sqrt{\frac{T}{2{\pi h\tau }_{0}}}$$

where $r_{\mathrm{plug}}\;$ is the plug flow radius, T is the torque, and h is the height of vane probe.

The Reiner–Riwlin equation taking into account plug flow is expressed by [24,25,26]:

(3) $$\omega =\frac{T}{4{\mu h\mu }_{p}}\left(\frac{1}{r_{1}^{2}}-\frac{1}{r_{\mathrm{plug}}^{2}}\right)-\frac{\tau_{0}}{\mu_{p}}\ln(\frac{r_{\mathrm{plug}}}{r_{1}})$$

where ω is the rotational speed and, $r_{1\;}$ is the vane probe radius.

To solve the Reiner–Riwlin equation by taking account of plug flow, an iterative nonlinear optimization method was used. Furthermore, the average shear rate at the inner vane probe was determined using the Reiner–Riwlin equation for the Bingham model as follows [25,27]:

(4) $$\dot{\gamma }=2T\frac{d\omega }{\mathrm{dT}}=\frac{T}{2{\pi h\mu }_{p}}\left(\frac{1}{r_{1}^{2}}-\frac{1}{r_{\mathrm{plug}}^{2}}\right)$$

**Figure 4:**
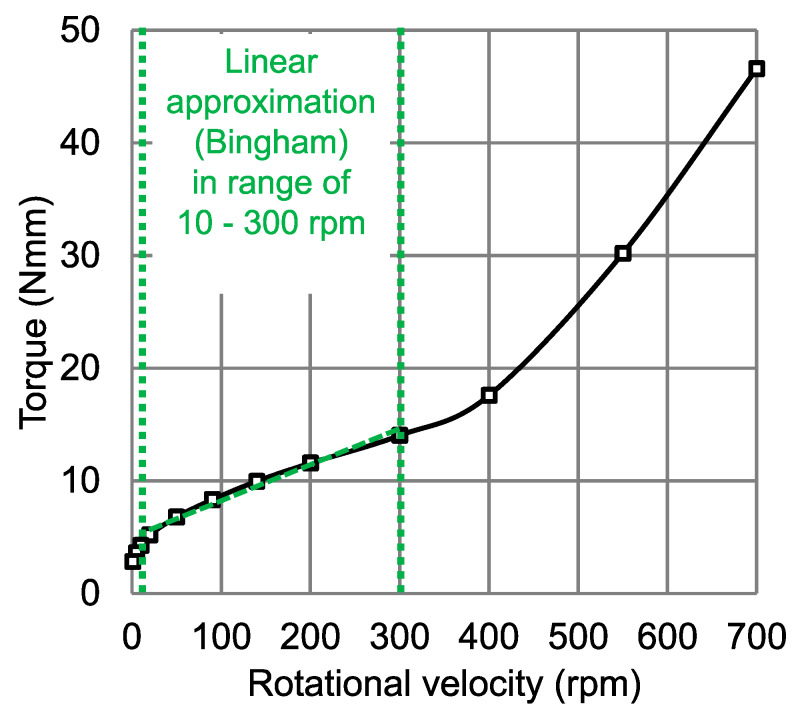
Typical results for torque as a function of rotational velocity, representative for all samples investigated with the Bingham approximation in the range of 10–300 rpm with regression coefficient (R^2^ = 0.998).

**Figure 5:**
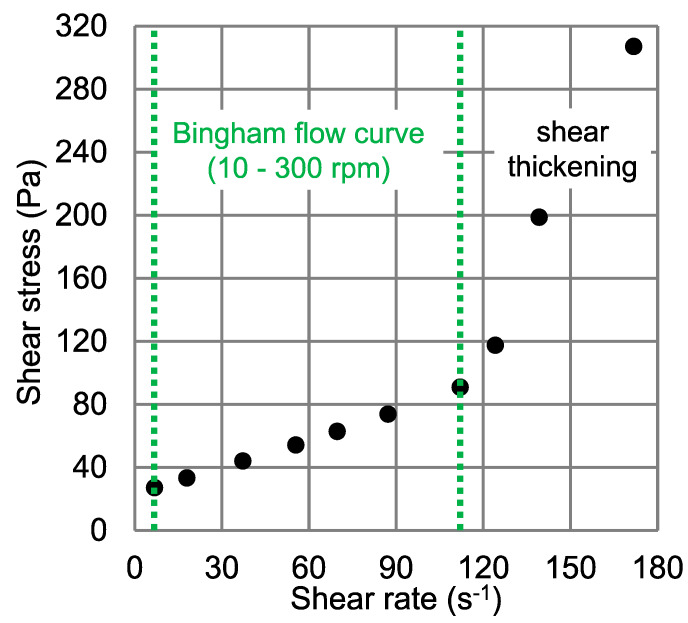
Average flow curve calculated for all measurements on cement paste.

Using Equation (4), an average shear rate at the inner vane probe between 10–300 rpm (range of linear approximation for the Bingham model, see Figure 4) was determined. With the help of the Bingham model, Equation (1), and the shear rates calculated from Equation (4) in each step, shear stress over shear rate curves, i.e., flow curve of the cement paste for the section with linear approximation, were obtained as shown in Figure 5.

#### 2.5.2. Shear Rate Calculation in the Shear Thickening Zone

To find the relationship between shear rate and microstructure, the rotational velocity in the shear thickening region must be converted to shear rate values as well. The Euler–Maclaurin series developed by Krieger and Elrod can be used to accurately calculate the average shear rate at the inner cylinder (at the vane probe in case of non-Newtonian fluids, respectively) in the case of fully sheared conditions in wide gap concentric cylinder geometries. With the ratio of the outer to the inner radius s ≤ 2 the expression is as follows [25,29,30,31,32]:

(5) $$\dot{\gamma }=\frac{\omega }{\ln s}\left(1+\ln s\frac{d\ln\omega }{d\ln\tau_{1}}+\frac{{\left(\ln s\right)}^{2}d^{2}\omega }{3\omega d{\left(\ln\tau_{1}\right)}^{2}}\right)$$

(6) $$\tau_{1}=\frac{T}{2{\pi r}_{1}^{2}h}$$

**Figure 6:**
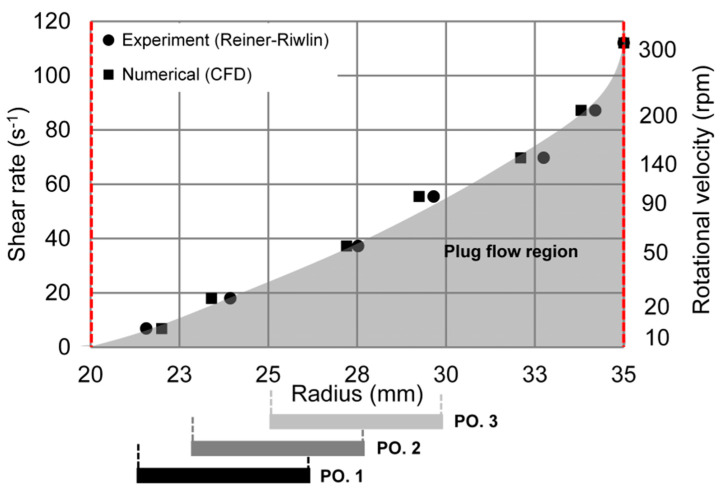
Plug flow radii over the shear gap determined by the analytical model and CFD simulations at different rotational velocities and its effect on different FBRM measurement zones.

In order to calculate shear rate values by Equation (5), the local gradient of the logarithmic plot of the shear stress $\tau_{1}$ (or torque T) versus rotational velocity ω is needed. The shear stress is calculated by Equation (6). As displayed in Figure 6, it can be concluded that fully sheared conditions in the case of the cement paste investigated here are given for rotational velocities of 300 rpm or larger, where the plug flow radius (shown as black circles) is located at the outer radius of the gap. In other words, the minimum rotational velocity needed to shear the entire material within the gap is 300 rpm. Hence, Equation (5) is valid to calculate the average shear rate on the vane probe for the shear thickening region.

## 3. Results and Discussion

### 3.1. Rheological Properties

The flow curve based on the experimental data (torque vs. rotational velocity) for a coupled rheometer-FBRM experiment is shown in Figure 4. The individual values were determined as average torque within the last 30 s of each rotational velocity step. The rheological tests were repeated three times in each radial position, yielding proper repeatability with the maximum standard deviation (SD) being 1.62 of the measured torque values.

Figure 5 shows the flow curve of the cement paste obtained by the analytical calculations based on the equations explained in Section 2.5. As can be seen, the cement paste investigated here exhibits at least two different rheological behaviors in different ranges of shear rate, i) a linear region (Bingham model in the range of 7–112 s^−1^) and ii) a shear thickening region (>112 s^−1^). In addition, a shear-thinning behavior of the cement paste may be observed at very low rotational velocities (<10 rpm). However, it should be mentioned that the lower region may not precisely represent shear thinning, only, as it can also be an artifact from a decreasing plug flow radius with decreasing rotational velocity, i.e., decreasing shear rate [24].

The rheological parameters were determined using the Bingham model with the linear approximation in the range of 10–300 rpm, as shown in Figure 4. The yield stress and plastic viscosity values calculated with the Reiner–Riwlin approach for the Bingham model were determined with an iterative nonlinear optimization method using the solver feature in Excel. For further details see [24,33]. The results are summarized in Table 2.

### 3.2. In-situ Chord Length Distributions

#### 3.2.1. Determination of Plug Flow Radius at Different Rotational Velocities

For the evaluation of the chord length distribution in dependence of the position of the FBRM probe, it is essential to know the shear rate distribution as well as regions of plug flow inside the gap. Therefore, the radial positions of the FBRM probe (numbered with 1, 2, and 3 in Figure 2) and the corresponding plug flow radii over the gap determined by the analytical model (Equation (2)) and CFD simulations in the range of 10–300 rpm are displayed in Figure 6. The average shear rate values shown in Figure 6 were calculated with the Reiner–Riwlin approach. The inner and outer walls of the setup are indicated by vertical red dashed lines.

The plug flow radii at each rotational velocity determined by CFD simulations are in very good agreement with the radii calculated by the analytical model. As shown in Figure 6, by decreasing the average shear rate at the vane probe, the plug flow radius shifts from the outer wall to the inner vane probe. The plug flow region of the cement paste investigated here as a function of average shear rates is highlighted with grey color. According to Figure 6, at 300 rpm, there is no plug flow in the outer radius of the gap. In other words, the entire material inside the shear gap is sheared (fully sheared condition). Hence, the minimum shear rate of 112 s^-1^ is needed for fully sheared conditions for the cement paste investigated.

However, in the context of the FBRM measurements, it is important to evaluate a possible plug flow radius at the position of the FBRM probe. When the plug flow radius arrives at the laser rotation diameter of the FBRM system, the fluctuations of the data detected by the FBRM probe are significantly increased. Thus, in our experiments, the chord length distributions detected by FBRM were only evaluated for rotational velocities higher than 140 rpm (70 s^−1^), as the plug flow radius at these shear rates does not reach the range of the laser rotation of the FBRM probe at any position.

#### 3.2.2. Effect of Shear Rates on Median Chord Length

**Figure 7:**
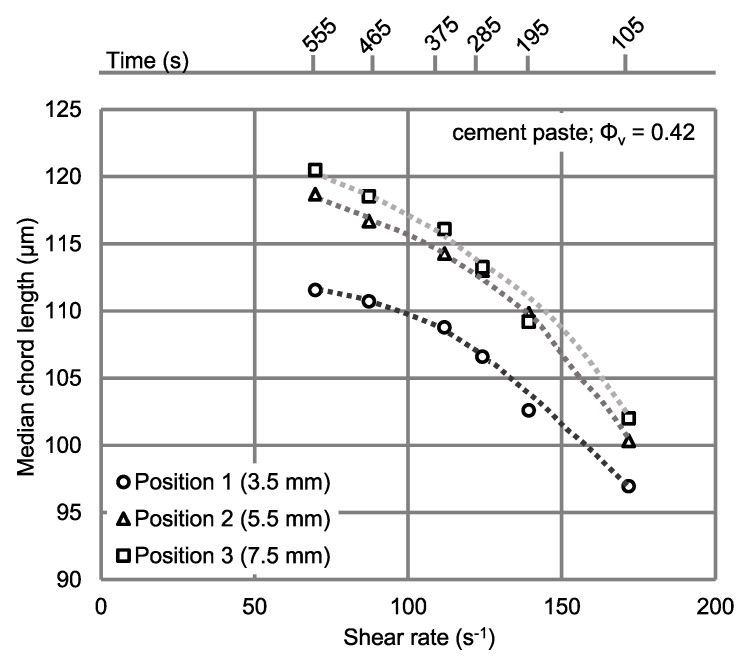
Averaged median chord length for different average shear rates in three different radial positions over the gap; the time shows the beginning of the experiments in the coupled FBRM-rheometer.

As for the torque data, the median chord length was determined as an average value within the last 30 s of each rotational velocity step. The results are shown in Figure 7.

As displayed in Figure 7, the median chord length (MCL) increases about 18 μm in all three positions when the average shear rate at the vane probe is decreased from 172 s^−1^ to 70 s^−1^. This is most likely due to increasing particle agglomeration with decreasing shear rates.

**Figure 8:**
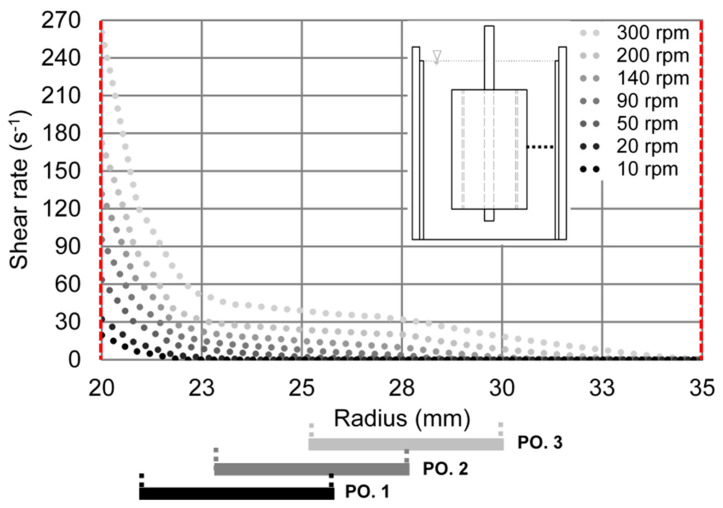
Shear rate distributions from CFD simulations within the gap at the middle height of the vane-in-cup rheometer (integration height of the FBRM system) for the cement paste investigated in the range of 10–300 rpm; inner and outer walls are marked with red dash lines.

Furthermore, MCL in position 1 (3.5 mm) is about 5 to 8 μm lower than in the other two positions (5.5 mm and 7.5 mm) for each shear rate, with a slight trend to higher differences for low shear rates. This effect can be attributed to the shear rate distribution within the gap. Figure 8 displays the actual shear rate distributions over the gap at the middle height of the vane probe simulated by ANSYS Fluent for different rotational velocities (10−300 rpm), taking into account the shear rate concentration at the tips of the vane probe.

According to the CFD simulations for the cement paste investigated here, there is a sharp decrease of the shear rate in the radial direction (over gap) in the vicinity of the vane probe, whereas the shear rate declines only slightly within the gap at larger distances. By reducing the applied rotational speed, the sharp decrease in shear rate occurs closer to the vane probe. It is important to mention that the shear rate in the immediate vicinity of the vane probe determined by the CFD simulation is higher than the average shear rate calculated by the Reiner–Riwlin approach, owing to the stress peaks at the vane probe tips. Due to the higher local shear rate at position 1, structural breakdown, i.e., particle dispersion is more pronounced as compared to the other two radial positions, resulting in lower MCL. Due to the small decrease in local shear rates over the radius between positions 2 and 3, the differences in MCL at the corresponding positions are also small, with the highest MCL values at position 3.

In addition to the local differences in structural breakdown affected by different local shear rates, shear-induced particle migration (SIPM) has to be taken into account as a source of differences in MCL at the three radial positions of the FBRM probe. SIPM, induced by radial shear rate variations, affects the movement of larger particles into regions of lower shear rates. As a consequence, the particle size distribution, as well as the solid volume fraction varies in radial direction as a function of shear rate, with larger particles migrating to regions of lower shear rates and smaller particles tending to accumulate in regions of higher shear rates. However, a clear distinction between the two effects on MCL, namely a) structural breakdown, i.e., agglomeration state, and b) shear-induced particle migration, requires further research.

#### 3.2.3. Effect of Shear Rates on Small Particles (≤ 2 μm)

**Figure 9:**
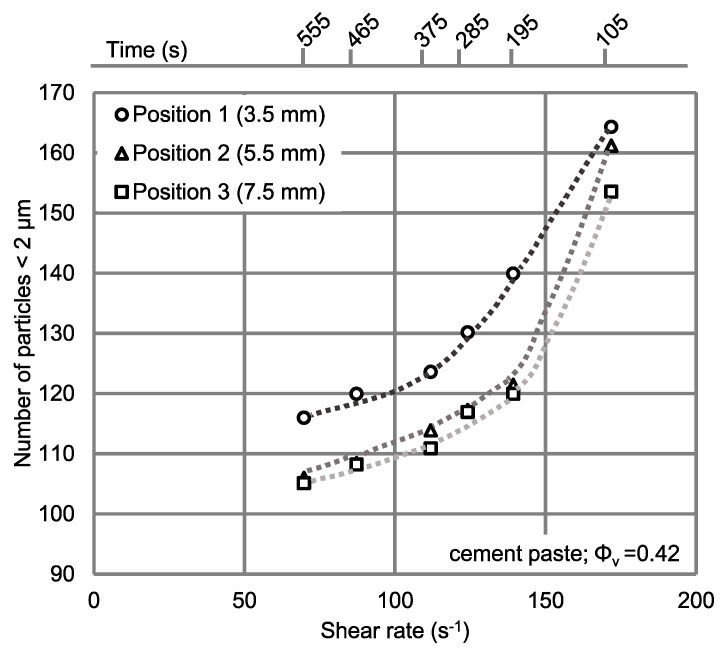
Particle numbers < 2 μm as a function of the average shear rate on the vane probe at different radial positions of the FBRM probe; time shows the beginning of the coupled FBRM-rheometer tests.

To gain additional information on the mechanisms effecting MCL variations over radial position and shear rate, the number of particles with chord length < 2 μm are displayed for three radial positions as a function of the average shear rate on the vane probe in Figure 9.

As expected, the number of small particles at radial position 1 is about 10% higher compared to the two other radial positions. As shown in Figure 9, the number of particles < 2 μm is reduced by about 31% in all radial positions when the average shear rate is decreased from 172 s^−1^ to 70 s^−1^. Moreover, the number of small particles is higher in position 1 compared to the two other positions because of the high local shear rates near the vane probe, showing disagglomeration and SIPM within the shear gap as a function of different shear rates.

## 4. Conclusions

The main objective of this study was to investigate the effect of shear history (average shear rate at the vane probe over time) and local shear rates (different radial positions within the gap) on the agglomeration state of cement paste. To identify the relationship between shear history and particle microstructure of a cement paste, a Focused Beam Reflectance Measurement (FBRM) system suitable to characterize particles in disperse systems was coupled with a wide gap vane-in-cup rheometer to measure chord length distribution and rheological properties, simultaneously. Based on the results, medium chord length increases by about 18 µm when the average shear rate on the vane probe is decreased from 172 s^−1^ to 70 s^−1^, resulting in larger particle agglomerates.

In addition, due to local shear rate differences at different positions of the FBRM probe within the shear gap (3.5, 5.5, and 7.5 mm), the median chord length increases with increasing radius by about 5 to 8 µm, indicating shear rate dependent differences in the local particle agglomeration state of the cement paste over radial direction. Furthermore, shear-induced particle migration must also be considered as an influencing factor on median chord length at different radial positions, effecting changes in local particle size distribution and, as a consequence, in local viscosity. In summary, the particle agglomerate structure at different positions within the shear gap, i.e., different local shear rates, is influenced by two main factors, a) shear rate dependent particle agglomeration and b) shear-induced particle migration.

## Figures and Tables

**Table 1:** Mixing procedure to prepare cement paste.

Process	Mixing Intensity	Duration
Dry homogenization of raw material	Level 1 (140 min^−1^)	60 s
Addition of water during mixing	Level 1 (140 min^−1^)	15 s
Mixing at lower speed	Level 1 (140 min^−1^)	45 s
Rest, manual homogenization	−	90 s
Mixing at higher speed	Level 2 (285 min^−1^)	60 s
Rest, manual homogenization	−	30 s
Mixing at higher speed	Level 2 (285 min^−1^)	120 s

**Table 2:** Bingham rheological parameters for cement paste.

Position of the FBRM Probe	No. Experiment	Bingham Model	
		Yield Stress (Pa)	Plastic Viscosity (Pa.s)
1	1	25.6	0.5
	2	24.1	0.5
	3	20.3	0.7
2	1	24.2	0.6
	2	20.4	0.7
	3	19.6	0.7
3	1	23.4	0.6
	2	21.9	0.6
	3	21.8	0.6
−	Average	22.4	0.61
